# Towards a Modernized Framework of Histology Teaching to Integrate Genetics: Pedagogical Perspectives for Oral Histology

**DOI:** 10.3390/genes16050512

**Published:** 2025-04-28

**Authors:** Camilla Sofia Miranda Kristoffersen, Camilla Elise Øxnevad Ziesler, Noora Helene Thune, Anna Tostrup Kristensen, Amer Sehic, Tor Paaske Utheim, Qalbi Khan

**Affiliations:** 1 Institute of Oral Biology, Faculty of Dentistry, University of Oslo, 0316 Oslo, Norwayamer.sehic@odont.uio.no (A.S.); utheim2@gmail.com (T.P.U.); 2 Department of Medical Biochemistry, Oslo University Hospital, 0424 Oslo, Norway; 3 Department of Plastic and Reconstructive Surgery, Oslo University Hospital, 0424 Oslo, Norway; 4 Department of Public Health and Sport Sciences, Inland Norway University of Applied Science, 2406 Elverum, Norway

**Keywords:** anatomy, genetics, morphology, oral histology, teaching strategies

## Abstract

Histology remains a cornerstone in medical and dental education, providing essential insights into tissue structure, function, and pathology. However, despite its foundational importance, interest in histology is declining, often due to outdated pedagogical methods, insufficient clinical context, and limited use of diverse teaching strategies. Modern health professionals require not only microscopic knowledge but also an understanding of the genetic mechanisms driving tissue development and disease. This paper critically evaluates current histology teaching strategies, identifying a gap in linking molecular genetics to tissue development, particularly in dental education. For instance, oral histology covers tooth development as a core subject yet often neglects the genetic foundations of odontogenesis. This disconnects risks undermining students’ ability to understand clinically relevant conditions, such as amelogenesis imperfecta, dentinogenesis imperfecta, molar incisor hypomineralization, and tooth agenesis—disorders where genetics play a key role. To address this, we propose a vertically integrated teaching model and a merged approach for teaching where several teaching methods, like flipped classrooms, team-based learning, and personalized digital tools, are designed for institutional curricula. Early pre-clinical exposure to genetic principles, revisited with clinical relevance in later years, can strengthen students’ appreciation of histology’s clinical value. This approach modernizes pedagogy, aligns with students’ preferences for digital learning, and ensures histology retains its central role in shaping competent healthcare professionals. Ultimately, developing multi-modal, genetics-integrated strategies is crucial to revitalizing histology education and fostering a deeper, clinically relevant understanding of human biology.

## 1. Introduction

Histology is a hands-on visual study that relies on microscopes and specialized staining techniques to identify and distinguish various cell types and tissue structures. Stained histological sections are examined at various magnifications, which allows an understanding of the organization of cells and how they shape tissues and organs. This examination not only reveals the developmental origins of tissues but also illustrates how they give rise to physiological structures and undergo pathological changes. Oral histology is the branch of histology that focuses on the microscopic study of the structure, organization, and function of oral tissues and their surrounding components [1].

It is alarming that students fail to perceive the relevance of general histology to their dental careers [2]. It is crucial that dental students receive histology education that maximizes their learning outcome and allows them to actively engage in their learning. However, studies show that the total hours for teaching histology have reduced overall [3]. To turn this trend, it can be beneficial to understand the subject’s historical changes into modern times, its methods of teaching, and assess the current state of histology education.

### 1.1. Historic Background

The emergence of histology, also known as microanatomy, as a discrete field within science was intrinsically linked to the innovation of optical technologies, particularly advancements in optical resolution. This dates back to the early 17th century, with the innovation of the first compound microscopes taking place across Europe. Notwithstanding several craftsmen and scientists aided the field of histology during its nascent phase, it was pioneered by four individuals [4]. The Englishman Robert Hooke (1635) used a simple compound microscope to explore biological material, thereby introducing the term “cell” into scientific discourse [5]. The Italian anatomist Marcello Malpighi (1628–1694) is credited to be the first to have examined the human body using a microscope. Through this, he revealed the capillaries, the missing link between arteries and veins [6], thereby confirming the circulation of blood [7]. The French pathologist Marie Francios Xavier Bichat (1771–1802) succeeded in defining the concept of “tissues” and differentiating 21 types [8], in fact, without the use of a microscope [4]. August Franz Josef Karl Mayer (1787–1865) coined the term histology in 1819, formed by the Greek words histos (tissue) and logica (study) [9].

During the 19th century, the limitations of early microscopes were reduced through advancements in optical resolution and image quality [10]. The British optician and physicist Joseph Jackson Lister (1786–1869) introduced the achromatic lens, which mitigated the phenomenon of chromatic aberration, thereby improving image clarity [11]. The German industrialists, opticians, and businessmen, Carl Zeiss (1816–1888), Ernst Karl Abbe (1840–1905), and Friederich Otto Schott (1851–1935) contributed to the compound microscope by introducing condenser and immersion objectives. These refinements inevitably led to the development of section preparation and staining techniques suitable for histological applications [4].

### 1.2. Significance of Histology Education in a Modern Genetic Context

Today, through histological analysis, clinicians do not only pierce into biological material to understand its microstructure but also place this understanding in a context of gaining insights into developmental disorders, the role of genetics, tissue regeneration, and disease mechanisms, which are essential for understanding cases encountered in clinical practice [12]. It gives a more integrated comprehension of the relationship between pathological features and clinical diagnosis as well as how genetic predispositions have been linked to a variety of oral health conditions [13].

For dental students, oral histology encompasses several key topics that facilitate the comprehension of microanatomy and the development of oral tissues [1]. This includes a detailed examination of the microstructure of the dental tissues, enamel, dentin, and pulp, each with distinct structural and physiological properties. Moreover, the oral mucosa and salivary glands are studied with regard to their histological organization and physiological functions, including their roles in immunity and saliva production. A key component of oral histology is the embryological development of these tissues—from the formation of the maxilla and mandible from the first pharyngeal arch to odontogenesis, with the interplay between ectomesenchymal and ectodermal cells [1].

Knowledge of tooth development provides a better understanding of how external factors may influence the stages of tooth formation and contribute to pathological conditions. It is essential for a dentist, as it provides a basis for prevention, diagnosis, and treatment of dental developmental disorders. This knowledge is particularly valuable when guiding patients to adopt appropriate behaviors during critical periods, as exemplified by the use of medication during pregnancy [14], which underlines the importance of instruction and information for pregnant patients. Further, physical trauma during childhood can interfere with the mineralization process of permanent teeth that have not yet reached full mineralization. This underscores the importance of children undergoing regular check-ups for early diagnosis and treatment of potential complications, like developmental enamel defects and hypoplasia, until and after the eruption of the permanent teeth [15].

The interaction between genetics and developmental disorders plays a significant role in various odontological conditions. This may be illustrated by the nature of amelogenesis imperfecta (AI) and dentinogenesis imperfecta (DI), which occur because of genetic mutations and can result in aberrant tooth structure [16]. Similarly, conditions such as cleft lip and palate result from complex genetic and environmental interactions, where early diagnosis and interdisciplinary treatment can be essential to optimize the patient’s prognosis [17]. Tooth agenesis, characterized by the absence of tooth development, presents another challenge for dentists due to genetic mutations [16], particularly in orthodontics and prosthodontics, where treatment planning must be tailored to the patient’s individual needs.

**Figure 1:**
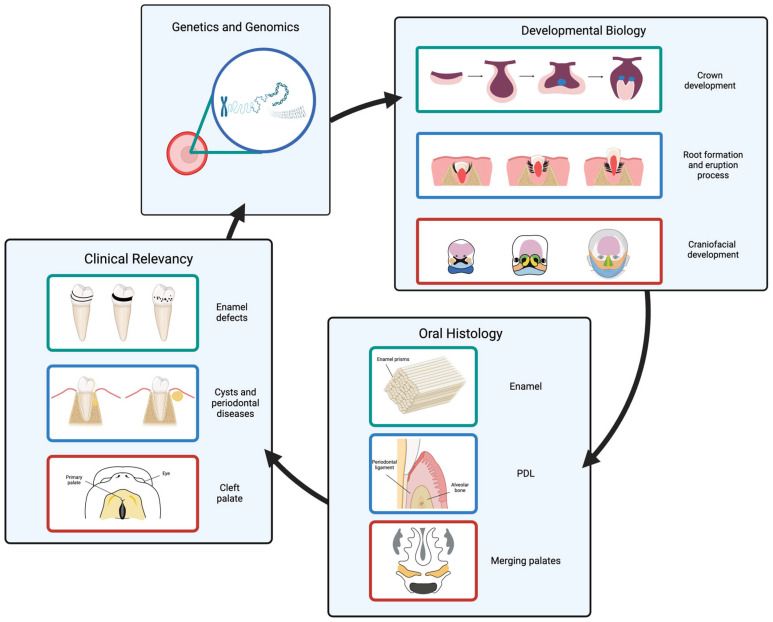
This figure shows how our knowledge of developmental biology and histology builds on our understanding of genetics and genomics. Furthermore, the relevancy of this knowledge has a direct high clinical value in the treatment caused by developmental disorders affecting oral tissues, teeth, and oral pathologies.

A more profound understanding of basic sciences provides clinicians with an opportunity to advise patients in the best way possible [12]. Both oral developmental biology and oral histology, when built upon the knowledge of their genetic background, become highly relevant for dental clinical aspects such as oral pathology, oral surgery, prosthodontics, orthodontics, and restorative dentistry (Figure 1).

Traditionally used teaching methods such as hands-on use of microscopy and in-classroom lectures are becoming obsolete, whereas more interactive courses and seminars are still widespread. Today, more modern approaches, such as virtual microscopy, group-based learning, quiz modules, flipped classrooms, and online lecturers, are implemented more widely. The aim of this overview is to assess available teaching methods to examine the effect on student motivation and learning outcomes, as well as to emphasize this to the teachers and administrative leaders of relevant institutions.

## 2. Overview of the Current Teaching Strategies

Teaching histology can be described as being taught using a two-component system, where the first component involves the didactic transfer of theoretical knowledge, typically delivered through lectures, self-directed learning materials, or even flipped classroom experience [4]. This didactic approach establishes a foundational understanding of histological concepts, where students must be involved in the memorization of cellular and acellular structures, cell and tissue types, and their developmental aspects as embryology, molecular biology, and genetics. The second component consists of active-learning laboratory sessions, where students engage with histological specimens through traditional light microscopy or virtual microscopy platforms. This active-learning approach fosters the development of recognition and visual interpretation skills, where students must analyze histological slides by identifying morphological structures made up of cells and tissues [4], combining them with the genetic and molecular basic understanding often embedded in the theoretical part. The first component, focusing on the theoretical part, and the second component, with emphasis on a practical hands-on approach, has a teaching effect of complementing each other.

These two modes of histology instruction reflect the dual-processing theory of learning, combining automatic, memory-based acquisition with analytical, higher-order reasoning skills. However, they overlap to teach the curriculum. A deep understanding of the methods and how they may effectively be combined to meet the modern student mass and a profession that stands steadily on its basic sciences is therefore warranted. In the following sections, we will examine each of the teaching methods that are applicable to histology teaching individually, analyzing their respective roles, advantages, and challenges in histology education. An overview of this is presented in Table 1.

### 2.1. Didactic Lectures

The didactic lecture approach is a traditionally used teaching method in which an instructor presents the subject matter in a one-directional manner, often using PowerPoint slides, blackboard, or other digital tools [24,58]. Well-established didactic approaches, such as traditional classroom lectures and histology laboratory work, are being replaced by a growing variety of innovative methods, including virtual microscopy, recorded lecture videos, and online learning modules [58,59]. The instructor usually serves as the primary source of knowledge by delivering a structured presentation, while the students assume a passive learning role as listeners with limited opportunities for interaction and discussion [22,23].

Didactic lectures offer several advantages within the framework of higher education. Foremost, the method is suited for efficiently conveying large quantities of information in a coherent way. The approach enables effective transmission of information, allowing the students to obtain fundamental knowledge within a condensed time frame. This elucidates why the method is commonly used in higher education, such as dentistry and medicine, where the subject matter must be efficiently conveyed due to its extensiveness [2]. Moreover, these lectures may serve as an overview of the subject matter, which can be advantageous prior to engaging in more active learning methods [24]. Traditional lectures, the majority of which utilize PowerPoint, have for an extensive period of time been the preferred method for delivering lectures, as they enhance the learning experience by the integration of key multimedia elements, such as visual cues and animations [60]. In addition, it is a cost effective learning method because it can accommodate large numbers of students and only requires few resources. Shared lectures also ensure standardization, providing all participants with a common baseline of knowledge. This is crucial in disciplines that require memorization of core concepts, including oral histology, as it guarantees that all students are exposed to the same key information [23]. Furthermore, the instructor ensures that key concepts are thoroughly explained, providing students with a clear and comprehensive foundational understanding. This approach helps students identify and retain the most essential concepts and details, preventing them from being overlooked amid the extensive volume of material covered in the curriculum [61].

Despite this, the approach of didactic lectures may promote passive learning wherein students acquire information without actively participating in the learning process. This can lead to a lower retention rate and limited critical thinking development, as students are not required to analyze or apply concepts in real-time [18,19]. Moreover, the unidirectional nature of such lectures constrains the opportunities for interaction between the teacher and students, making it challenging to seek clarification or engage in discussions [20]. The absence of interactivity may create a disconnect between pre-clinical instruction and practical patient care [24]. Additionally, the absence of personalized learning poses a challenge, as this approach is designed for broad acceptability rather than accommodating individual learning styles. Consequently, some students may encounter difficulties in grasping complex concepts, which hinder necessary conceptual understanding, while others may find the content insufficient in rigor [21]. Finally, didactic lectures tend to focus on the transmission of knowledge as opposed to skill development, which can reduce their efficacy in such fields that necessitate hands-on experience and critical analytical reasoning [18]. Thus, such an absence of knowledge application and interactivity may create a distance to the dental profession [62].

### 2.2. Light Microscopy

Light microscopy has long been an essential tool in the teaching of dental histology and has been a fundamental component of early pre-clinical dental education. The approach provides students with a teaching experience that is hands-on in handling and examining tissue structures at a microscopic level. By using the microscope and handling physical glass slides, students develop technical skills, such as focusing techniques, using correct objective lenses and contrast adjustments, and, most importantly, a dimensional understanding of histological features [63]. In earlier times, students also learned how to operate a light microscope with detailed instructions in manuals on microscope design and the physics that lay behind it. Students were also provided staining protocols, so they could participate in the staining, thereby underscoring the hands-on learning method. Learning histology through developing an individual slide collection benefits the understanding of the biological material at hand, the processing of it, and the variability found in the sections. However, this approach has slowly shifted as students began receiving loan-ready-made collections of permanent glass slides, arguing for the financial aspects and a greater focus on microscopic analysis [4].

Light microscopy is a valuable tool in histology education, offering detailed visualization of cellular morphology, tissue organization, and pathological changes. This hands-on approach fosters a deeper understanding of oral histology and its clinical relevance by allowing students to examine real specimens directly. Additionally, the use of individual glass slide collections allows students to appreciate the natural variability of biological specimens and tissue preparations, reinforcing their observational and analytical skills. Beyond enhancing theoretical knowledge, light microscopy promotes critical thinking and diagnostic abilities, which is helpful for clinical decision-making in dental practice [25].

Despite its advantages, the role of light microscopy in dental education is increasingly debated due to advancements in digital and virtual microscopy. Traditional microscopy is time consuming and requires specialized equipment, maintenance, and supplies, making it less accessible [26]. Another limitation is that multiple sectioned tissue samples are often needed to observe all relevant structures, as a single slide may not capture every detail of interest, limiting comprehensive tissue analysis [27,28]. Glass slides, while essential for hands-on learning, are prone to fading and breakage, necessitating constant replacement, which imposes a continuous financial burden on educational institutions [4]. Furthermore, the quality of student glass slide collections can vary, and certain rare specimens may not be accessible to all learners, making the sharing of slides among students essential [29]. The method of traditional light microscopy is gradually becoming outdated, as newer technologies offer more efficient alternatives for histological learning. These challenges highlight the need to balance traditional and modern approaches in histology education.

### 2.3. Drawing Microscopy Images

The task of drawing microscopy images is a pedagogical technique that engages students or professionals in creating hand-drawn representations of histological sections, whether viewed through a light microscope or a virtual platform [64]. The method has been used extensively over time, as it was once the means of documenting histological sections prior to the advent of photography [4].

As an interactive learning approach, drawing offers several cognitive advantages that enhance the comprehension and retention of complex biological structures. Engaging actively in the drawing process enhances memory retention by facilitating deeper cognitive processing, as one must retrieve and examine detailed histological structures while producing a visual representation [30,31]. Additionally, it fosters a deeper understanding of the arrangement and intricacies of the tissue structure, providing a more profound comprehension of their functional and morphological interconnections [32]. Even though studies assessing the impact of learning histology via drawing in comparison to traditional microscopy revealed no significance between the groups, students reported augmented retention and a better grasp of histological concepts in clinical applications [65]. By actively creating their own representations, students are likely to reveal potential knowledge gaps and engage in contemplative thinking, which has proved to enhance engagement and critical thinking [33].

Despite its advantages, this learning approach also presents certain limitations. A primary drawback is the considerable time investment it demands. Producing accurate and detailed histological illustrations is time consuming, which may restrict the scope of content that can be covered [30]. This time constraint is particularly challenging in accelerated academic environments, where students must balance multiple academic responsibilities simultaneously [66]. Furthermore, students’ artistic abilities present a limitation to this method as those with less-developed drawing skills may struggle to accurately depict microscopic structures, potentially shifting the focus towards the technical aspects of drawing rather than the core curriculum content [34]. Additionally, the variable quality of the visual depositions might result in misrepresentations of anatomical and functional structures, causing misconceptions if the drawings are not closely supervised by an instructor [35,36].

### 2.4. Virtual Microscopy

The traditional histological teaching methods discussed in previous sections have evolved in recent years and have been adapted to align with the increasingly digital and virtual learning environment of the present [4]. Virtual microscopy initially emerged as a method for viewing static digital images but has now transformed into a tool that enables recording, storage, and dissemination of high-resolution scanned micrographic images [67]. Additionally, it allows students to access entire slides or specific regions from their own electronic devices, as well as to zoom in and out for detailed examination, facilitating a more flexible and comprehensive learning experience.

The use of these high-resolution scanned micrographic image files has proven to yield positive results [29], and reports show that virtual slides have solved a number of problems encountered with light microscopy. These challenges have been addressed based on feedback from users, with comments highlighting several advantages of virtual microscopy. Responses included the following: “Doesn’t hurt my eyes. Clear image always in focus”, “Much better. Faster to use, therefore more efficient”, “Less dizzy, less fuss, quicker learning”, and “More convenient to use, and about the same quality image, if not better” [37]. These statements reflect the improved user experience and efficiency associated with virtual microscopy. Virtual microscopy contributes to a more equitable histology education, as all students have access to the same high-quality slide materials. Additionally, virtual microscopy creates greater opportunities for team-based learning, as it facilitates collaboration, encourages the formulation of questions, and promotes discussion among students [4,41].

Virtual microscopy is a highly preferred alternative to traditional methods among students [26]. However, despite its growing popularity, there are potential drawbacks to this approach. As the field continues to shift away from optical microscopy and toward virtual microscopy, transitioning histology courses entirely to a virtual microscopy lab may introduce limitations and challenges that require careful consideration. Discontinuing the use of traditional light microscopy in histology education would eliminate opportunities for learners to experience variations in slide quality and the appearance of tissues or organs. It has been questioned whether mastering the use of a traditional light microscope remains an essential skill for medical professionals and should therefore continue to be incorporated into pre-clinical education [4]. Although most practicing dentists do not use microscopes, it may be beneficial to briefly expose students to conventional microscopes and glass slides during their general medical education, allowing them to better understand the basic principles of histology and the concept behind virtual images [26].

### 2.5. Group/Team-Based Learning

Progressive dental and medical curricula are aiming to make biomedical education more student-centered by incorporating group-based learning [38,39]. This instructional approach enables students to collaborate in groups, fostering a deeper and more comprehensive understanding of the subject matter. Initially, students are provided with an overview of the subject matter, thus expecting them to master the basic facts and concepts of it, explicitly enabling them to actively participate in the subsequent activities [68]. Within the field of oral histology, systematic examination and analysis of specimens, along with problem-solving and peer-teaching, are particularly relevant [69]. The use of virtual microscopy is well suited for group-based learning, as all participants can simultaneously observe the histological virtual microscope images on the same screen. This ensures that all participants make the same observations and remain engaged, thus commencing scientific discussion [41,68].

Group- or team-based learning offers several pedagogical advantages that elevate comprehension, engagement, and overall academic attainment. Research has shown that this method enhances knowledge retention and academic performance, particularly when integrated with virtual microscopy [40]. Students who employed a team-based approach achieved significantly higher test scores in comparison to students who followed traditional instructional formats [41]. Small group discussions foster active engagement, peer interaction, and cooperative problem-solving, not only enhancing the understanding of the subject matter but also replicating the collaborative nature of clinical practice [42]. Such interactivity facilitates higher-order cognitive processing, entailing students to evaluate, apply, and synthesize histological concepts rather than simply committing structural details to memory [4]. Furthermore, instant feedback from peers and teachers enables students to clarify misconceptions right away, reinforcing accurate interpretations [41]. Lastly, group-based learning advances students’ motivation and accountability, as students are more inclined to participate actively when they are responsible for contributing to a collective understanding [42].

Although group-based learning in oral histology offers significant pedagogical advantages, it also presents several challenges that can reduce its effectiveness if not properly designed and implemented. One primary concern is the imbalance in student participation, where more extroverted and academically strong students may dominate discussions, while less assertive students may adopt a passive role, leading to disparities in learning outcomes [42]. Additionally, group-based learning tends to be more time consuming than conventional didactic lectures, as it necessitates active student engagement through discussion, peer teaching, and collaborative problem-solving [43]. Moreover, this educational activity requires additional faculty resources, as instructors must be present to facilitate discussion, if necessary, provide guidance, and ensure that students remain aligned with the course objectives. Without adequate guidance, students may reinforce incorrect interpretations of histological features, potentially leading to knowledge gaps if not detected [42].

### 2.6. E-Learning Elements

Students nowadays are often described as “digital natives” or the “YouTube generation”, reflecting their familiarity with technology-based learning [44]. This suggests that integrating e-learning elements into dental education could be an easy approach to implement if student satisfaction is considered a major factor.

E-learning elements in dental histology education encompass a wide range of digital resources, including e-books, websites, e-learning platforms, social media, podcasts, online tutorials, Massive Open Online Courses (MOOCs), and mobile applications [4]. E-learning resources offer several pedagogical advantages by providing broad access to diverse educational materials, including videos, e-books, and interactive modules. These platforms support flexible, self-paced learning, allowing students to tailor their study strategies to individual needs. Importantly, e-learning enhances student engagement through interactive content, multimedia explanations, and structured opportunities for collaborative learning. Additionally, digital tools facilitate the visualization of complex concepts, thereby improving comprehension. Educators also benefit from dynamic, multimedia platforms that support interactive teaching and are easily updated to reflect current knowledge. Notably, the integration of e-learning platforms has been associated with improved learning outcomes across various educational settings [44,45,46].

Despite their potential advantages, the integration of digital resources into dental histology education remains inconsistent, with most tools being used only sporadically [4]. Given the variability in quality, it is essential to critically assess e-learning components, as some may contain inaccuracies or misinformation. This concern is particularly relevant when social media platforms are employed as educational tools for medical and dental students, where ensuring the reliability and accuracy of content is paramount. Careful evaluation of information sources and verification of author credibility are necessary to maintain educational integrity and prevent the dissemination of erroneous material [44].

### 2.7. Quiz Module and Gamification

Quiz module and gamification is a self-directed learning method that incorporates interactive assessments and game-like elements to foster student engagement, enhance motivation, and improve knowledge retention [47]. The method of the quiz module enables students to engage with questions at the desired pace, receive immediate feedback, and adjust the level of difficulty [70]. Gamification incorporates elements such as points, levels, achievement badges, and leaderboards to cultivate a competitive and cognitively stimulating learning environment that enhances motivation and engagement [48]. In the context of oral histology, such self-teaching methods can be utilized to enhance students’ ability to identify and differentiate histological structures and pathological alterations. This approach can be implemented in various forms, ranging from traditional methods such as flashcards and multiple-choice questions to more digitalized solutions, including digital flashcards (Anki and Quizlet), game-based quiz platforms (Kahoot!), and virtual microscopy games (Histopoly) [49,50,51].

The incorporation of quiz modules and gamification offers several advantages that enhance student engagement, knowledge acquisition, and self-efficiency. One of the primary advantages is the flexibility it provides, allowing students to engage with the subject matter at their own pace and revisit content as needed [47]. This autonomy fosters a more comprehended understanding and encourages students to take greater responsibility for their own learning, which is particularly advantageous in disciplines that necessitate the integration of theoretical foundations with clinical applications, such as histology and pathology [71]. Additionally, the active and continuous nature of this learning approach has been shown to augment knowledge retention and conceptual understanding [72]. The immediate feedback provided in self-guided modules further enhances learning by enabling students to identify misconceptions in real-time [52]. Incorporating game-like elements improves student motivation by fostering a sense of accomplishment and encouraging sustained engagement [73]. Additionally, changes in dental and medical curricula necessitate the development of learning strategies that decrease student and instructor contact while preserving effective pedagogical methods [47]. This self-directed learning approach is both cost effective and scalable, as the module can be efficiently distributed across multiple cohorts without adding to faculty workload [71].

While quiz modules and gamification offer several advantages in oral histology education, they also present certain limitations. A major drawback is the potential overemphasis on extrinsic motivation, where students focus more on earning points of rewards rather than developing a comprehended understanding of histological concepts [53]. Additionally, these methods may not cater to all learning styles, as some students may require hands-on experience with real microscopy and instructor guidance to fully grasp complex tissue structures [4]. Furthermore, quiz modules often assess recognition rather than critical thinking, which may not adequately prepare students for clinical applications where diagnostic reasoning is essential [54].

### 2.8. Flipped Classroom and Online Lecturers

The flipped classroom is a blended learning model that combines online technology with instructor-led active learning experiences. While few studies have evaluated its effectiveness for pre-clinical medical students, several articles suggest it could be an ideal approach for this setting [74]. According to existing literature, the “flipped classroom” is an educational approach that “reverses the lecture and homework elements of a course”. [75]. In this model, students complete what was traditionally considered “classwork”, such as learning basic definitions and explanations, at home. Conversely, activities that were previously assigned as “homework”, including problem-solving exercises, are now conducted during class time [55]. Additionally, the flipped classroom is a method shifting from traditional didactic lectures to a model where students access online course materials before class. During in-person sessions, learning is reinforced through small-group peer teaching and team discussions guided by faculty [44].

The flipped classroom model has gained recognition as an effective alternative to traditional lectures, with several benefits promoting active learning and student engagement. By providing foundational course materials online in advance, students could engage with the content at their own pace before attending in-person sessions [56]. As a result, students come to class prepared for interactive sessions focused on problem-solving, critical thinking, and applying knowledge. This approach allows faculty to dedicate class time to more in-depth discussions and activities, enhancing student engagement and deeper understanding [55]. Additionally, this method enhanced long-term retention and recall of information and aligned with the upper levels of Bloom’s taxonomy, which emphasizes critical thinking and application of knowledge [76].

While the flipped classroom approach offers several benefits, it also presents certain challenges that must be addressed for effective implementation. It requires more from the students. Both self-motivation and self-discipline become important factors when the workload for students increases [24]. Additionally, students may express concerns about the reliability of information provided by their peers during such collaborative learning sessions. Another key challenge is the need for faculty training to effectively implement this teaching method, as both technological and logistical issues must be accounted for [56]. It is therefore important to acknowledge these limitations and emphasize the importance of proper guidance and evaluation to ensure the method’s success.

## 3. Conclusions

Histology remains a critical part of several education areas, given its nature of providing fundamental insight into tissues, their interaction, and their pathology. Although macroscopic understanding is seen as more clinically relevant, without the microscopic basis, our knowledge would be both insufficient and rudimentary. It is therefore alarming that the interest in it is fading away, both from the curriculum and the student’s minds. In a survey based on 100 histology teachers, 75% responded that some reforms were needed [77]. Another survey among students found that 35% wished for improvement in teaching style, while 28% proposed a reduction in the histology curriculum [35]. More specifically for oral histology, dental students view the subject as both more difficult and less relevant than medical students [2]. These adverse trends may be attributed to several factors, including an overemphasis on content-heavy instruction, insufficient integration of clinical context, limited use of diverse pedagogical strategies beyond traditional lectures, and a lack of emphasis on the teaching staff’s professional background [77]. Therefore, an overview of the method of teaching, in order to decide what is working as beneficial for teaching and learning, along with what is warranted for future professions such as medicine and dentistry, is of utter importance.

This paper evaluates current pedagogical strategies so that we may obtain a clearer view of the changes needed to improve the interest in the field of oral histology, the methods that need to be implemented to achieve this, and the importance of basic sciences with clinical relevancy. There is a clear understanding that digital tools are often more welcomed by students than the teachers. The reason for this may be that teachers do not have the same familiarity with it. Teachers may be skeptical of the ready-made digital tools that can be found online. It is time consuming to detail-check resources and hence demotivating. However, as digital tools are both wanted and proven important in teaching, a third option should be considered optimal. Developing your own digital tools that are personalized and fitted for the institution’s curriculum may solve this issue. Our previous studies on tooth morphology demonstrated that strategies that merged flipped classroom and team-based learning were found both interesting and enhancing for learning [57]. A similar approach might be fruitful for histology also.

We also found that very few reports of histology teaching linked directly to the genetic basis of this field. For example, in oral histology tooth development is taught as a central part of the dental curriculum. However, embryological development that is driven by molecular genetics is often separated or little focused on. Understanding the genetics of tooth development is crucial for dental students, as dental genetic disorders have an important clinical value. AI, DI, Molar Incisor Hypomineralisation, agenesis, supernumerary teeth, morphological variances, etc. are all examples of the clinical picture a modern dentist must understand. However, adding more oral genetics to the already halting subject of histology might be considered too ambitious a task to take on. Instead of improving the interest, one might be afraid that it may end up complicating the subject even further and hence demotivating the students furthermore. However, our understanding is that if the subject is lightly introduced early, usually as a pre-clinical part, it should be easier to repeat in later years with more clinical aspects introduced. Here, we may be able to successfully implement the concept of vertical teaching, where a greater emphasis and priority of time is laid upon integrating the teaching of basic sciences and the clinical aspects of it together [78].

Normally in dental schools, the curriculum instructs dental students to complete the same basic courses as medical students during the first two years before separating and moving on to three years of practical dentistry education [78]. At the University of Oslo, oral histology (along with oral genetics and developmental biology) is taught for a duration of 5 weeks after the separation and before the dental clinical part. We propose cutting down the hours in the pre-clinical parts and integrating these parts into clinical years. For example, the recalling and applying of knowledge related to AI is performed best when observing patients clinically at pediatric dentistry sessions. This may affect the outline of the curriculum, but staff, recourses, estimated time per course, and teaching materials can remain stable.

**Figure 2:**
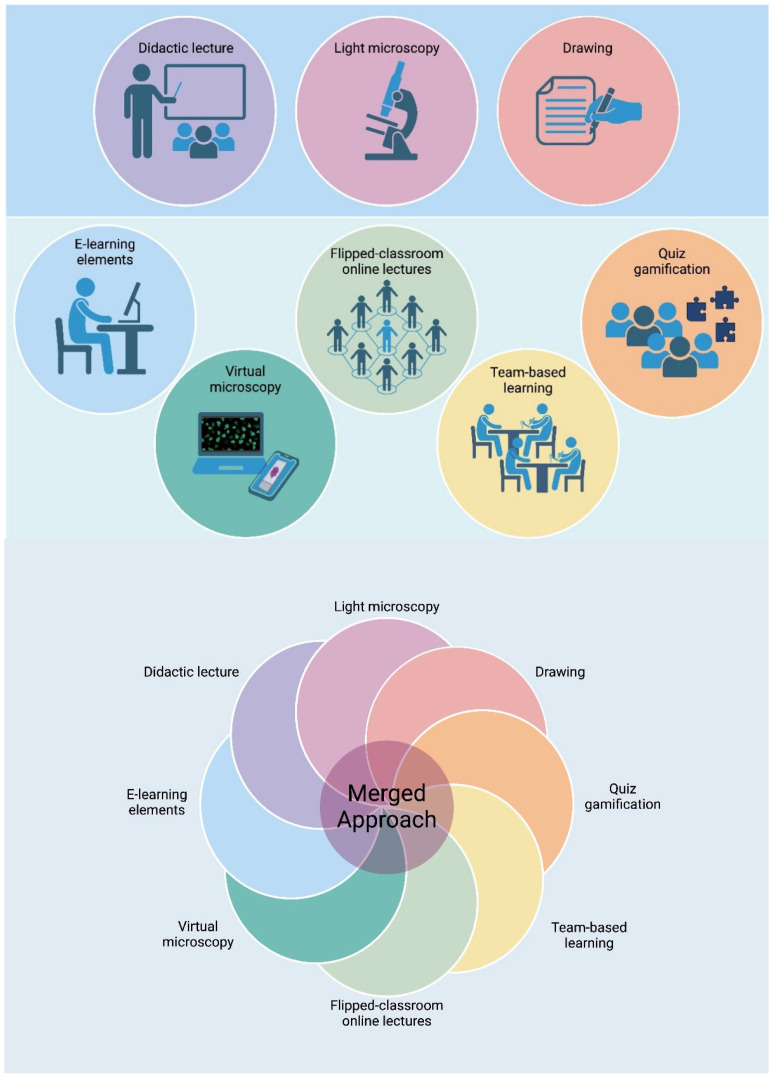
This figure shows both the traditional (**upper bar**) and the more modern types of teaching methods (**lower bar**) that are suitable for dental histology teaching. We propose a teaching approach that successfully merges several methods in order to engage and deliver several aspects of histology.

In conclusion, instead of deciding on using one or a few tools for teaching histology, one should imply a method that merges different teaching methodologies (Figure 2). In our opinion, by merging several techniques into the same course of histology, we can obtain a deeper understanding of the interdisciplinary related to histology, such as tissue development, pathologies, and genetics.

The dental curriculum should be built on vertical teaching that only introduces the “heavy basic sciences” in a pre-clinical part. Later, in the clinical part, it should be repeated in a specifically clinical context. This way, both the pedagogical skills of teachers will remain up to date, as well as important basic science aspects of dentistry, that are currently perceived as dull or unmotivating, stay vitalized as clinically valid. In the future, greater emphasis should be put on supporting teaching staff in expanding their methodological repertoire. If feasible, institutions should aim to develop an integrated, multi-modal approach that addresses both curricular demands and the constraints of time and resources faced by different institutions.

## Figures and Tables

**Table 1:** Drawback and advantages of different methods of teaching applicable to histology teaching.

Methods of Teaching	Drawbacks	Advantages	References
Didactic lectures	May promote passive learning Limited room for critical thinking Limited room for hands-on skills development Challenging to seek clarification or engage in discussions Designed for broad acceptability rather than accommodating individual learning styles	Students assume a learning role as active listeners Efficiently conveying large quantities of information in a coherent way Cost-effective: It can accommodate large numbers of students and only requires few resources Instructor can ensure that key concepts are thoroughly explained	Freeman et al., 2014 [18] Mentzer et al., 2023 [19] Sirikumpiboon 2014 [20] Prince 2004 [21] Hortsch 2024, [22] Obrez et al., 2011 [23] Johnson et al., 2015 [2] Overskott et al., 2024 [24] Hortsch 2023 [4]
Light microscopy	Time-consuming Resource-consuming Less accessible Limiting tissue analysis on a single slide, as it may not capture every detail of interest Variations in the quality of glass slide collections	Active learning in hands-on approach Promotes critical thinking Enhances diagnostic abilities Helpful in clinical decision-making	Lallier 2014 [25] Alotaibi et al., 2016 [26] Blake et al., 2003 [27] Bloodgood & Ogilvie, 2006 [28] Hortsch 2023 [4] Weaker and Herbert, 2009 [29]
Drawing microscopy images	Time-consuming Too much focus on drawing skills Room for misrepresentations of anatomical and functional structures	Enhance the comprehension and retention of complex biological structures Enhances memory retention by facilitating deeper *cognitive* processing Active learning in hands-on approach Room for revealing potential knowledge gaps Promotes critical thinking	Balemans et al., 2016, [30] Cogdell et al., 2011 [31] Heideman et al., 2017 [32] Kotze et al., [33] Horne et al., 2024 [34] García et al., 2018, [35] Campos-Sanches et al., 2012 [36]
Virtual microscopy	Less experience of variations in slide quality Lower understanding of the basic principles of histology	Less time-consuming Standardized High-quality slide materials Individual learning (flexible)	Kumar et al. [37] Hortsch 2023 [4] Alotaibi et al., 2016 [26]
Group/Team-based learning	Imbalance in student participation More time-consuming Additional faculty resources to ensure adequate guidance	Student-centered Enhances knowledge retention Higher test scores and academic performance Interactive engagement Peer interaction Cooperative problem-solving—replicating the collaborative nature of clinical practice Advances students’ motivation and accountability	Khalil et al., 2013 [38] Jurjus et al., 2018 [39] Sander & Golas 2013 [40] Goldberg & Dintzis, 2007 [41] Bloodgood 2012 [42] Hortsch 2023 [4] Xue et al., 2021 [43]
E-learning Elements	Sporadically/inconsistent integration in the curriculum Quality can vary Some may contain misinformation Extra attention needed to ensure reliable and accurate educational content	Diverse learning materials Individual learning (flexible) Interactive content may exist Multimedia explanations	Lone et al., 2018 [44] Bogacki et al., 2004 [45] Maggio et al., 2012 [46] Hortsch 2023 [4]
Quiz and Gamification	Emphasis on recognition rather than critical thinking for clinical diagnostic Too much focus on points/rewards	Enhance student engagement and motivation Self-teaching options Flexibility Active learning Identification of misconceptions in real-time Cost-effective	Thompson et al., 2017 [47] Pawar et al., 2024 [48] Kalleny 2020 [49] Mishall et al., 2023 [50] Marcos et al., 2025 [51] Badyal et al., 2019 [52] Hanus & Fox 2015 [53] Rowe et al., 2012 [54]
Flipped classroom and online lecturers	Needs conversion from a lecture room to discussion rooms Extra attention needed to ensure proper organization and logistics Requires high level of student self-motivation	Active learning and student engagement Individual learning (flexible) Emphasizes critical thinking and application of knowledge	Lone et al., 2018 [44] Gilliland, 2017 [55] Park & Howell 2015 [56] Markholm et al., 2024 [57]

## Data Availability

Not applicable.
